# Design of a Novel Gene Therapy Construct to Achieve Sustained Brain-Derived Neurotrophic Factor Signaling in Neurons

**DOI:** 10.1089/hum.2017.069

**Published:** 2018-07-01

**Authors:** Andrew Osborne, Aiden X.Z. Wang, Alessia Tassoni, Peter S. Widdowson, Keith R. Martin

**Affiliations:** ^1^ John van Geest Centre for Brain Repair, Department of Clinical Neurosciences, University of Cambridge, Cambridge, United Kingdom; ^2^ Quethera Ltd., Babraham Research Campus, Cambridge, United Kingdom; ^3^ Cambridge NIHR Biomedical Research Centre, Cambridge, United Kingdom; ^4^ Eye Department, Addenbrooke's Hospital, Cambridge, United Kingdom; ^5^ Wellcome Trust—MRC Cambridge Stem Cell Institute, University of Cambridge, Cambridge, United Kingdom.

**Keywords:** brain-derived neurotrophic factor, tropomyosin-related receptor kinase-B, gene therapy, adeno-associated viral vector

## Abstract

Brain-derived neurotrophic factor (BDNF) acting through the tropomyosin-related receptor-B (TrkB) is an important signaling system for the maintenance and survival of neurons. Gene therapy using either recombinant adeno-associated virus (AAV) or lentiviral vectors can provide sustained delivery of BDNF to tissues where reduced BDNF signaling is hypothesized to contribute to disease pathophysiology. However, elevation in BDNF at target sites has been shown to lead to a downregulation of TrkB receptors, thereby reducing the effect of chronic BDNF delivery over time. A novel gene sequence has been designed coding both the ligand (BDNF) and the TrkB receptor in a single transgene separated by a short viral-2A sequence. The single transgene is efficiently processed intracellularly *in vitro* and *in vivo* to yield the two mature proteins, which are then independently transported to their final cellular locations: TrkB receptors to the cell surface, and BDNF contained within secretory vesicles. To accommodate the coding sequences of both BDNF and TrkB receptors within the narrow confines of the AAV vectors (4.7 kb pairs), the coding region for the pro-domain of BDNF was removed and the signal peptide sequence modified to improve production, intracellular transport, and secretion of mature BDNF (mBDNF). Intracellular processing and efficacy was shown in HEK293 cells and SH-SY5Y neuroblastoma cells using plasmid DNA and after incorporating the TrkB-2A-mBDNF into an AAV2 vector. Increased BDNF/TrkB-mediated intracellular signaling pathways were observed after AAV2 vector transfection while increased TrkB phosphorylation could be detected in combination with neuroprotection from hydrogen peroxide–induced oxidative stress. Correct processing was also shown *in vivo* in mouse retinal ganglion cells after AAV2 vector administration to the eye. This novel construct is currently being investigated for its efficacy in animal models to determine its potential to progress to human clinical studies in the future.

## Introduction

Mature brain-derived neurotrophic factor (mBDNF) is a 14 kDa protein member of the neurotrophin family of growth factors.^1,2^ Brain-derived neurotrophic factor (BDNF) binds to its cognate tropomyosin-related receptor-B (TrkB), inducing receptor dimerization and subsequent multiple tyrosine trans-phosphorylation.^3–5^ TrkB phosphorylation is necessary to mediate pro-survival signaling through the phosphoinositol-3-kinase (PI3K), phospholipase C-γ1, and MAP kinase (MAPK) pathways.^6–9^

BDNF is initially produced as a 32 kDa protein containing a signal peptide, proBDNF domain (which has been proposed to facilitate protein folding^10,11^), and a mature segment (mBDNF).^12^ Processing occurs either intracellularly or extracellularly to yield proBDNF and mBDNF, with most proBDNF being cleaved into mBDNF in neurons.^13–15^ It is mBDNF that acts as a high affinity ligand at TrkB receptors, while the larger proBDNF protein has been shown to modulate pro-apoptotic pathways via the p75^NTR^ low-affinity neurotrophin receptor.^16–20^

BDNF levels have been hypothesized to be reduced in Alzheimer's disease, Parkinson's disease, and Huntington's disease.^21–23^ BDNF depletion has also been suggested to play a role in glaucoma,^24^ with evidence for impaired retrograde BDNF transport from the brain to the retina demonstrated in experimental models of ocular dysfunction.^25–27^

At present, recombinant BDNF delivery has not been successful therapeutically, possibly due to the poor ability of the protein to cross the blood–brain barrier when administered intravenously and the need for frequent dosing due to rapid protein breakdown.^28^ Gene therapy using recombinant viral vectors offers a way to produce therapeutic proteins continuously following a single administration. Recombinant adeno-associated virus (AAV) vectors have been widely used in human gene therapy clinical studies. Advantages of AAV include a lower risk of genome integration and higher multiplicity of infection (MOI) than lentiviruses.^29^

Available evidence suggests that overexpression of BDNF can cause tachyphylaxis of the biological response through the downregulation of TrkB and its subsequent degradation,^30^ as well as replacement of functional TrkB receptors by truncated TrkB receptors lacking kinase activity.^31–33^ This study therefore aimed to design a vector that could deliver both BDNF and its receptor TrkB, with both proteins targeted to the correct cellular compartments.

The aim of the program was to design a gene therapy construct that was capable of sustained BDNF/TrkB signaling and which would overcome the normal tachyphylaxis response of this pathway, thereby providing long-term cellular survival against a broad range of pathophysiologic insults.

## Methods and Materials

### Plasmid and vector constructs

Codon optimization of DNA sequences was performed using an online tool,^*^ and DNA blocks were synthesized by Integrated DNA Technologies, Inc. (Skokie, IL) or GenScript (Piscataway, NJ). Cloning was performed using standard molecular biological approaches. DNA plasmids were scaled up in SURE competent cells (Agilent Technologies, Santa Clara, CA) to achieve quantities of 500 μg endotoxin free, transduction quality DNA. AAV2 vectors were produced by Vigene Biosciences (Rockville, MD). Vector particles were liberated following the freeze–thaw of HEK293 cells transduced with plasmid DNA, followed by iodixanol gradient ultracentrifugation and de-salting, and were suspended in phosphate-buffered saline (PBS; Thermo Fisher Scientific, Waltham, MA). Titers were confirmed by Vigene Biosciences through quantitative polymerase chain reaction using primers recognizing the ITR regions.

### Construct design

To incorporate two transgenes into a single AAV2 vector requires careful selection of the regulatory units and cargo design. By conventional means, fitting the BDNF ligand and TrkB receptor into an AAV2 vector would not be possible (Supplementary Fig. S1; Supplementary Data are available online at www.liebertpub.com/hum). In designing the constructs, regulatory elements were selected to ensure maximal transgene expression while utilizing the minimum number of base pairs (bp). A strong synthetic CAG promoter was opted for, frequently used to drive high levels of gene expression in mammalian expression vectors.^34,35^ Modifications were made to the cytomegalovirus (CMV) element, chicken beta-actin promoter (CBA), and rabbit beta 1-globulin component to provide a minimal 678 bp CAG promoter (Supplementary Fig. S1B).

A modified woodchuck hepatitis virus posttranscriptional regulatory element (WPRE) (247 bp) was also selected described by Choi *et al.*,^36^ which boosts gene expression in a variety of cells and species.^37^ The shortened WPRE element saves 345 bp, has the same properties as the full-length 592 bp version, and removes the contaminating X-protein sequence that has been implicated in tumorigenesis.^38^ Finally, a 224 bp simian virus 40 late polyadenylation signal (polyA) was selected to improve mRNA stability and boost translational efficiency.^39^ These choices would ensure optimal transgene expression, but further modifications would be necessary to fit both ligand and receptor within the narrow confines of an AAV2 vector (4.7 kbp).

### Cell culture

HEK293 and SH-SY5Y cells were cultured in poly-L-lysine (10 μg/mL; Sigma–Aldrich, St. Louis, MO) coated six-well plates or 13 mm poly-L-lysine coated glass coverslips in Dulbecco's minimum essential medium (DMEM) containing 10% fetal bovine serum (FBS), and 1% penicillin/1% streptomycin (1% Pen/Strep) until 80% confluent. The medium was then exchanged for DMEM (no additives), and cells were transduced with 4 μg plasmid DNA and 4 μL/mL lipofectamine (Thermo Fisher Scientific) for 24 h at 37°C. Vectors, diluted in sterile PBS were added directly to DMEM (no additives) at a final concentration of 1 × 10^10^ viral particles (vp)/mL and incubated for 48 h. The BDNF antagonists ANA-12^40^ and Compound-G^41^ were purchased from Sigma–Aldrich and Princeton Bio Molecular Research, Inc. (Monmouth Junction, NJ), respectively, and administered at 10 μM 1 h after transduction. Rat C6 glioma cells were cultured in uncoated 96-well plates in 100 μL of DMEM, 10% FBS, 1% Pen/Strep until 50% confluent. The medium was then exchanged for 100 μL of DMEM (no additives) containing mBDNF (cat. #ab9794; Abcam, Cambridge, United Kingdom) at concentrations between 25 and 200 ng/mL, or 100 μL of transduced HEK293 cell incubation medium. The rat C6 glioma cells were then incubated for a further 72 h and proliferation assessed using the CellTiter 96^®^ AQueous One Solution Cell Proliferation Assay (Promega, Madison, WI).

### *In vivo* intravitreal injection of AAV2 vector

Adult male C57BL/6 mice (Charles River Laboratories, Wilmington, MA) were anesthetized with an intraperitoneal injection of ketamine (50 mg/kg) and xylazine (10 mg/kg) and given topical 1% tetracaine eye drops prior to injection in accordance with the British Home Office regulations for the care and use of laboratory animals, the UK Animals (Scientific Procedures) Act (1986), and the Association for Research in Vision and Ophthalmology's Statement for the Use of Animals in Ophthalmic and Visual Research. AAV2 vector (2 μL), diluted in sterile PBS, was drawn up into a 5 μL glass syringe (Hamilton Company, Reno, NV) with a fine metal micropipette with a tip diameter of 30 μm and a tip length of 2.5 mm. Using an operating microscope, AAV2 vector was injected through the sclera into the vitreous of the eye approximately 3 mm posterior to the superior-temporal limbus. Care was taken to avoid penetration of the lens or damage to the vortex veins during the intravitreal injection. Injections were given slowly over 1 min to allow diffusion of AAV2 vector suspension. Animals were culled via CO_2_ inhalation 3 weeks (21 days) later, ensuring stable transgene expression. AAV2 vectors were injected at 1 × 10^10^ vp/eye.

### Secreted BDNF measurement

Secreted BDNF was measured in culture medium 24 h after transduction. Medium was centrifuged to remove debris and measured using a commercial human mature BDNF enzyme-linked immunosorbent assay (ELISA) kit (Sigma–Aldrich) or human proBDNF ELISA kit (Biosensis, Thebarton, South Australia). BDNF concentration was determined by comparing samples to freshly made BDNF standards.

### Western blotting

Protein extraction was performed by washing cells in cold PBS and lysing in Lysis-M reagent containing cOmplete Mini Protease Inhibitor (Roche, Basel, Switzerland) and phosphatase inhibitors (Thermo Fisher Scientific). Similarly, retinal tissue was excised from the eye globe, frozen on dry ice, and digested in lysis buffer. Cells were homogenized on ice for 20 min and then centrifuged at 9500 *g* for 10 min to isolate the soluble cell extract. Protein concentration was determined using a bicinchoninic acid protein assay (Thermo Fisher Scientific). Equal quantities of protein were loaded into wells of Bis-Tris gels (10% and 4–12% NuPAGE Novex; Thermo Fisher Scientific) and examined by Western blotting. Primary antibodies included polyclonal anti-BDNF antibodies (cat. sc-546; Santa Cruz Biotechnology, Inc., Santa Cruz, CA; used at 1:500 dilution for cells, 1:200 for retinal tissue), polyclonal anti-TrkB antibodies (cat. ab33655; Abcam; 1:2,000 cells, 1:500 retinal tissue), polyclonal anti-pTrkB (Y515) antibodies (cat. ab109684; Abcam; 1:750), polyclonal anti-phospho-Akt (Ser^473^) (p-Akt; cat. 9271; Cell Signaling Technology, Danvers, MA; 1:300), polyclonal anti-phospho-ERK1/2 (Thr^202^/Tyr^204^) (p-ERK1 or p-ERK2; cat. 4370; Cell Signaling Technology; 1:600), polyclonal ERK1/2 (cat. 4695; Cell Signaling Technology; 1:1,000), polyclonal Akt (pan; cat. 4691; Cell Signaling Technology; 1:1,000), polyclonal anti-GFP antibodies (cat. 4695; Invitrogen; Carlsbad, CA; 1:1,000 cells; cat. ab290; Abcam; 1:3,000 retinal tissue), or polyclonal beta actin (cat. 4970; Cell Signaling Technology; 1:1,000) incubated overnight at 4°C in 5% dried skimmed milk in PBS with 0.2% Tween20 (Sigma–Aldrich). Primary antibodies were visualized with horseradish peroxidase conjugated anti-rabbit secondary antibody (Vector Laboratories, Peterborough, United Kingdom; 1:8,000) and signal detection using ECL Prime (GE Healthcare, Little Chalfont, United Kingdom) and an Alliance Western blot imaging system (UVITEC Ltd., Cambridge, United Kingdom). BDNF expression was normalized to beta actin, p-TrkB to t-TrkB, and p-Akt, p-ERK1, and p-ERK2 were normalized to t-Akt, t-ERK1, and t-ERK2.

### Immunocytochemistry

Cells were washed twice in PBS and fixed for 30 min in 4% paraformaldehyde in PBS at room temperature. After three more washes, cells were blocked and permeabilized by incubation in 5% normal goat serum, 3% bovine serum albumin (BSA), and 0.3% Triton X-100 in PBS for 60 min at room temperature. Primary antibodies included BDNF (cat. sc-546; Santa Cruz Biotechnology, Inc.; 1:300) or TrkB (cat. ab33655; Abcam; 1:500) diluted in blocking solution and incubated overnight at 4°C. Staining was revealed using secondary anti-rabbit antibodies conjugated to Alexa Fluor (AF) 647 (Invitrogen; 1:1,000) for 2 h at room temperature. Cell nuclei were also counterstained with 1 μg/mL of DAPI (Thermo Fisher Scientific; 1:8,000). Cells were further washed three times before being mounted with fluorSave™ reagent (Calbiochem^®^/EMD Chemicals, Inc., Gibbstown, NJ) prior to imaging. Imaging was carried out using a 20 × objective and a Leica DM6000 epifluorescence microscope (Leica Microsystems, Wetzlar, Germany) or a Leica SP5 confocal microscope (Leica Microsystems) equipped with a 40 × oil objective using a 3.00 × digital zoom and 0.5–0.8 sequential scanning z-step interval.

### Immunohistochemistry

Eyes for immunohistochemistry were either imaged as retinal flat mounts or sections. For sections, eyes were dehydrated in 30% sucrose in PBS at 4°C for 24 h and embedded in silicon molds containing optimal cutting temperature compound (Sakura Finetek, Torrance, CA). Eyes were then frozen on dry ice before being sectioned at 13 μm through the dorsal-ventral/superior-inferior axis of the retina onto superfrost plus slides (VWR International, Lutterworth, United Kingdom) using a Bright OTF 5000 cryostat (Bright Instruments, Luton, United Kingdom). Retinal flat mounts were prepared following dissection of the posterior eye structure and removal of the lens. The retinas were gently dissociated from the underlying retinal pigment epithelium, flattened, and post fixed for 30 min in 4% paraformaldehyde in PBS prior to staining. Retinal flat mounts or sections were washed in 0.5% Triton X-100 in PBS and frozen at −80°C for 10 min to permeate the nuclear membrane and improve antibody permeation before blocking in 10% normal donkey serum, 2% BSA, and 2% Triton X-100 in PBS for 60 min at room temperature. Retinal ganglion cells (RGCs) were counterstained with antibodies against Brn3A to visualize RGCs (cat. sc-31984; Santa Cruz Biotechnology, Inc.; 1:200), BDNF (Santa Cruz Biotechnology, Inc.; 1:300), or TrkB (Abcam; 1:500) diluted in blocking solution and incubated overnight at 4°C. Secondary antibodies included anti-goat AF555 (Invitrogen; 1:500) and anti-rabbit AF647 (Invitrogen; 1:1,000) with DAPI for 2 h at room temperature. Retinas were imaged using confocal microscopy at 40 × or 63 × objective using a 1.50–3.00 × digital zoom.

### Hydrogen peroxide–induced SH-SY5Y cell death

Forty-eight hours after SH-SY5Y cell transfection, the medium was exchanged for fresh DMEM (no additives). Hydrogen peroxide (H_2_O_2_; Thermo Fisher Scientific) was diluted in filtered water (to a concentration of 0.1 or 1.0 mM) and added at an equal volume to wells or plates for an additional 24 h. Filtered water served as a vehicle control. For TUNEL staining (product # G3250; Promega), cells were washed three times in PBS and immersed in TUNEL equilibration buffer for 10 min. The TUNEL reaction mixture was made per the manufacturer's protocol and was added to cells for 1 h at 37°C. The reaction was stopped by incubating in 1 × standard citrate solution (SCS) for 15 min. Cell nuclei were counterstained with DAPI. Cells were imaged using a 20 × objective and counted by hand by an investigator blinded to the treatment groups.

### Statistics

All data are represented as the mean ± standard error of the mean (SEM). Statistical analysis was performed using Student's *t*-test for unpaired groups or analysis of variance followed by Bonferroni modified *t*-tests for multiple comparisons.

## Results

### Improving mBDNF production and secretion

To aid construct size and to reduce activation of pro-apoptotic pathways, the coding sequence for BDNF needed to be modified. The first stage included removing the coding for the proBDNF domain from wild type (wt)-S proBDNF, which when expressed via gene therapy can have a detrimental effect on neuronal survival,^16,18,19^ and adjoining the endogenous 18-amino acid signal sequence [MTILFLTMVISYFGCMKA] to the mBDNF sequence (wt-S mBDNF). Expressing mBDNF without the pro-domain (wt-S mBDNF) produced a similar overall amount of intracellular BDNF to wt-S proBDNF, although approximately 50% of BDNF remained uncleaved in the wt-S proBDNF group (Supplementary Fig. S2A and B). No uncleaved proBDNF could be detected following HEK293 cell transduction with wt-S mBDNF (Supplementary Fig. S2A and B) nor was proBDNF detected in the culture medium (Supplementary Fig. S2C), although there was also reduced mBDNF in the media (data not shown).

**Figure 1.**
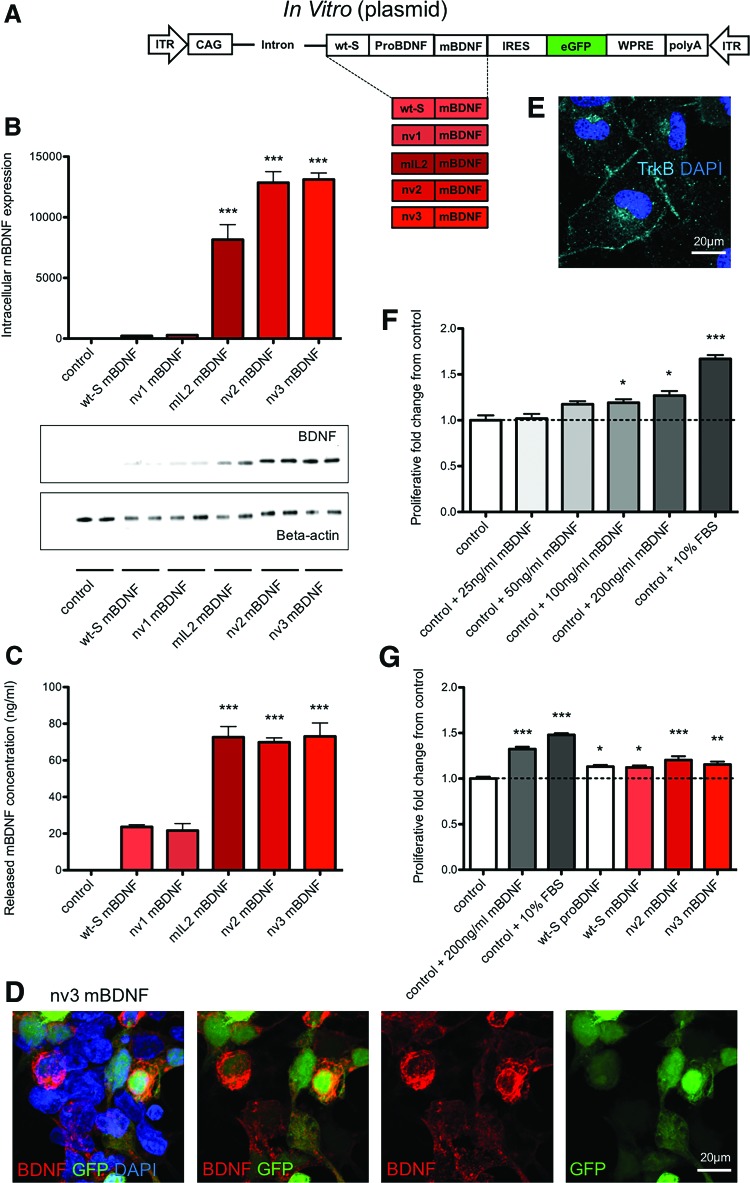
Optimizing the brain-derived neurotrophic factor (BDNF) sequence by removal of the pro-domain and modifying the signal peptide to improve BDNF production and secretion. **(A)** Schematic of the constructs tested. **(B)** Modifying the signal peptide could improve BDNF production over endogenous levels (*n* = 4); ***p < 0.001 compared to wt-S mature BDNF (mBDNF). **(C)** Secreted, mBDNF was increased after signal peptide modification (*n* = 3–4); ***p < 0.001 compared to wt-S mBDNF. **(D)** Expression of BDNF was observed in transduced nv3 mBDNF, green fluorescent protein (GFP)-positive, HEK293 cells. **(E)** Expression of the tropomyosin-related receptor-B (TrkB) receptor was detectable on naïve rat C6 cells. **(F)** Addition of recombinant mBDNF could enhance C6 proliferation over 72 h (*n* = 2–4). **(G)** Supernatant from transduced HEK293 cells could increase C6 proliferation after an additional 72 h in culture (*n* = 4); *p < 0.05, **p < 0.01, and ***p < 0.001 compared to controls. Color images available online at www.liebertpub.com/hum

Second, novel signal peptide sequences were designed to maximize mBDNF levels by ensuring optimal correct protein folding during production and increased secretion in the absence of the preceding proBDNF domain, which is known to interact with the intracellular protein, sortilin to traffic the complex from the Golgi complex to dense core secretory vesicles (Fig. 1A). This was accomplished by increasing the initial number of basic amino acids at the N-terminal portion and increasing the proportion of lipophilic amino acid sequences in the middle section, as described by Zhang *et al.*^42^ Varying signal peptide sequences were devised ranging from 21-amino acids contained in nv1 mBDNF [MKRRVMIILFLTMVISYFGCMKA] and nv2 mBDNF [MRRMQLLLLTMVISYFGCMKA] and 18 amino acids in nv3 mBDNF [MRILLLTMVISYFGCMKA]. Coding for the modified 20 amino acid interleukin-2 (mIL-2) signal peptide [MRRMQLLLLIALSLALVTNS]^42^ was also synthesized and incorporated into plasmid mIL-2 mBDNF as a reference for the novel signal peptides.

HEK293 cells were transduced with the plasmids wt-S mBDNF, nv1 mBDNF, mIL-2 mBDNF, nv2 mBDNF, or nv3 mBDNF, and the level of BDNF-immunoreactivity was measured in cell homogenates and levels released into the incubation medium (Fig. 1B and C). Switching the signal peptide to the modified IL-2 sequence (mIL-2 mBDNF) or a modified novel BDNF sequence (nv1 mBDNF, nv2 mBDNF, or nv3 mBDNF) significantly increased intracellular mBDNF expression, indicating that these alternative signal sequences may improve protein synthesis. The novel signal peptide sequences contained in nv2 mBDNF and nv3 mBDNF produced the greatest level of BDNF protein in the cell lysates (Fig. 1B). Assessing the cell culture medium also revealed mIL-2 mBDNF, nv2 mBDNF, and nv3 mBDNF generated an enhanced amount of secreted mBDNF, which was approximately three to four times greater than the endogenous sequence in wt-S mBDNF (Fig. 1C).

Immunocytochemical analysis of the BDNF immunoreactivity in HEK293 cells following transduction revealed a pattern of staining indicative of BDNF storage in secretory vesicles (Fig. 1D). Similar staining was observed after transduction with all constructs, and removal of the proBDNF domain did not appear to affect the intracellular localization of BDNF (Supplementary Fig. S2D and E). Similar patterns of staining were also visible in mouse RGC layer neurons transfected, with AAV2 vectors expressing wt-S proBDNF or wt-S mBDNF, indicating BDNF was also being successfully translated in the absence of the proBDNF domain *in vivo* (Supplementary Fig. S2F and G). To confirm further that the mBDNF produced and released from HEK293 cells had correctly folded and was functional, conditioned medium was collected 24 h after transduction and applied to cultures of rat C6 glioma cells. Rat C6 cells were confirmed to express the TrkB receptors (Fig. 1E), and previous studies have shown they respond to BDNF by proliferating.^43^ It was confirmed that application of recombinant mBDNF significantly increased rat C6 glioma cell number, which was apparent at concentrations of 100 and 200 ng/mL (Fig. 1F). In response to conditioned media from HEK293 cells transduced with plasmids wt-S mBDNF, nv2 mBDNF, and nv3 mBDNF, there was a significant increase in cell proliferation, consistent with the effect seen with recombinant mBDNF. Novel constructs also displayed comparable levels of proliferation to wt-S proBDNF (Fig. 1G).

### Testing dual constructs containing the viral-2A peptide linker

Internal Ribosome Entry Site (IRES) linkers allow transcription of a second transgene from a single construct. However, the IRES element is large (574 bp), and the efficiency of IRES-dependent translation differs between cells and tissues. Additionally, the expression level of the downstream gene is often significantly lower than that of the preceding upstream gene in bicistronic vectors.^44,45^

**Figure 2.**
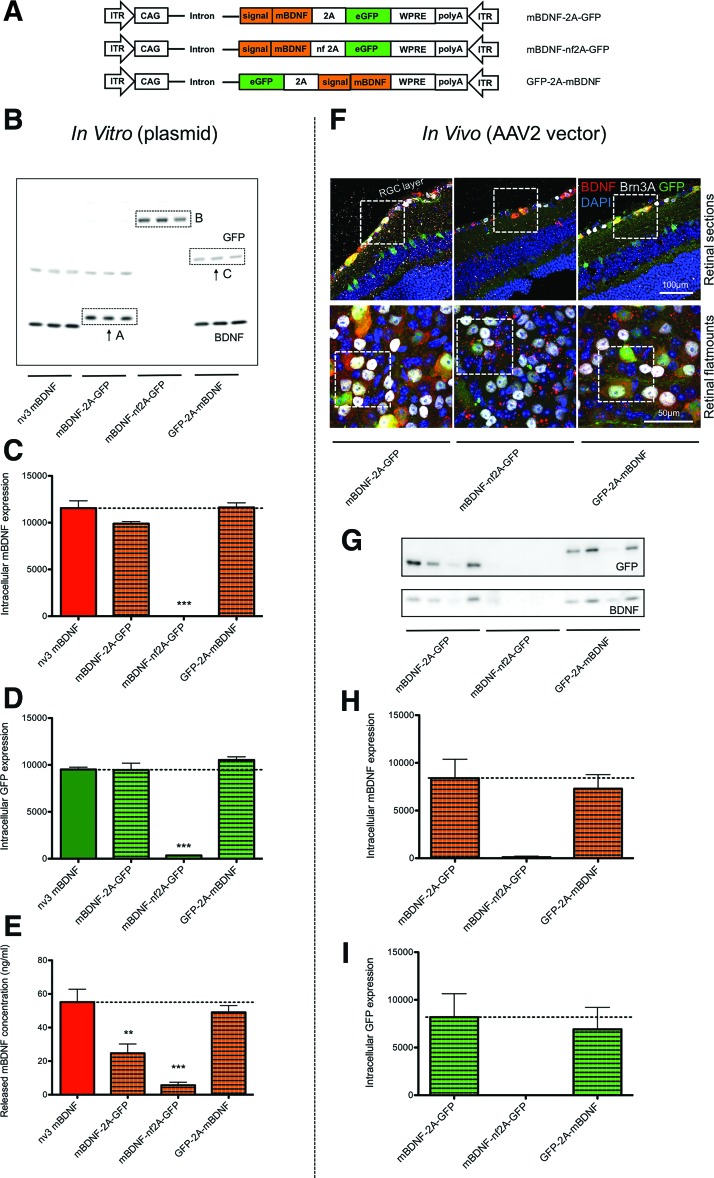
Cleavage using a 2A linker to liberate two separate proteins, GFP and mBDNF, *in vitro* (HEK293 cells) and *in vivo* (mouse retina). **(A)** Schematic of the orientation of the transgenes and whether the 2A linker was functional (2A) or non-functional (nf 2A). **(B–D)** Correct cleavage and separation of proteins GFP and BDNF *in vitro* 24 h after transduction compared to nv3 mBDNF with a functional 2A linker (*n* = 3); **p < 0.01 and ***p < 0.001 compared to nv3 mBDNF. **(E)** Release of BDNF from HEK293 cells transduced with plasmids containing a functional 2A linker was detected in culture medium. **(F)** Correct cleavage and separation of GFP and BDNF *in vivo* in the mouse retina 3 weeks after adeno-associated virus (AAV) serotype 2 vector transfection (*n* = 4). GFP and BDNF could be detected in separate locations within Brn3A+ retinal ganglion cells (RGCs), further detailed in Supplementary Fig. S3. **(G–I)** GFP and BDNF were separated *in vivo* with AAV2 vectors containing a functional 2A linker in lysates from the mouse retina (*n* = 4). All AAV2 vectors used at 1 × 10^10^ viral particles (vp)/eye. Color images available online at www.liebertpub.com/hum

Therefore, a foot-and-mouth viral-2A peptide sequence (63 bp) was chosen that can liberate two mature proteins from a single transgene under the control of a single promoter. Three plasmids were designed to test the efficacy of cleavage using a 2A linker separating mBDNF and enhanced green fluorescent protein (eGFP; Fig. 2A). The mBDNF-2A-GFP construct contained the reading frame for nv3 mBDNF followed by eGFP. GFP-2A-mBDNF contained the same elements as mBDNF-2A-GFP, except that the mBDNF and eGFP order was reversed, and GFP-nf2A-mBDNF was identical to mBDNF-2A-GFP, except that the coding for the last four amino acids of the viral-2A peptide sequence [NPGP] was replaced by four alanines [AAAA], which produces a non-functional cleavage site.^46^ Production of mBDNF and eGFP by the three plasmids was compared to nv3 mBDNF, which contained coding for both mBDNF and eGFP separated by the IRES element.

Western blotting revealed a band for mBDNF at 13–14 kDa with nv3 mBDNF and GFP-2A-mBDNF (Fig. 2B). The BDNF band was also observed with mBDNF-2A-GFP, with a slight shift in molecular weight due to the mBDNF having the additional amino acid associated with the remaining N-terminal of the viral-2A linker (see Fig. 2B, band A). GFP-nf2A-mBDNF revealed a high molecular weight band of around 40 kDa, corresponding to the uncleaved large precursor protein of both GFP and BDNF (Fig. 2B, band B). All the other plasmids produced an eGFP band around the 27 kDa range, except for GFP-2A-mBDNF, which displayed a slightly heavier band (see Fig. 2B, band C) corresponding to the eGFP with the remaining viral-2A peptide tag attached. Quantification of the BDNF and eGFP immunoreactivity showed that there were equivalent levels of protein produced by nv3 mBDNF, mBDNF-2A-GFP, and GFP-2A-mBDNF (Fig. 2C and D).

Comparable levels of secreted mBDNF were detected in the culture medium from nv3 mBDNF and GFP-2A-mBDNF transduced cells (Fig. 2E). Significantly less mBDNF was detected in medium from mBDNF-2A-GFP, possibly due to the attachment of the 2A tag to the BDNF C-terminal, while minimal secreted BDNF was obtained from GFP-nf2A-mBDNF. These data suggest that the optimal confirmation for maximal BDNF release from the cell is to position the BDNF downstream from the coding region for the first protein (eGFP), as the remaining viral-2A peptide sequence, which remains attached to the C-terminal of BDNF appears to attenuate protein release partially from HEK293 cells.

To test whether the viral-2A peptide sequence could liberate two mature proteins *in vivo*, mice were intravitreally injected with AAV2 vectors coding for mBDNF-2A-GFP, GFP-nf2A-mBDNF, or GFP-2A-mBDNF, and 21 days later, retinas were examined for cleavage efficacy. Similar to *in vitro* results, AAV2 mBDNF-2A-GFP and AVV2 GFP-2A-mBDNF revealed separate bands corresponding to mBDNF and GFP, with the expected shift in molecular weight due to the attachment of the 2A tag. Expectedly, bands for mBDNF and GFP were absent in retinas transfected with AAV2 GFP-nf2A-mBDNF (Fig. 2G–I). Immunohistochemistry also confirmed functional cleavage of the 2A linker demonstrated by separate intracellular locations of GFP and BDNF within RGC layer neurons (Fig. 2F and Supplementary Fig. S3).

### Creation of a TrkB-2A-BDNF construct

**Figure 3.**
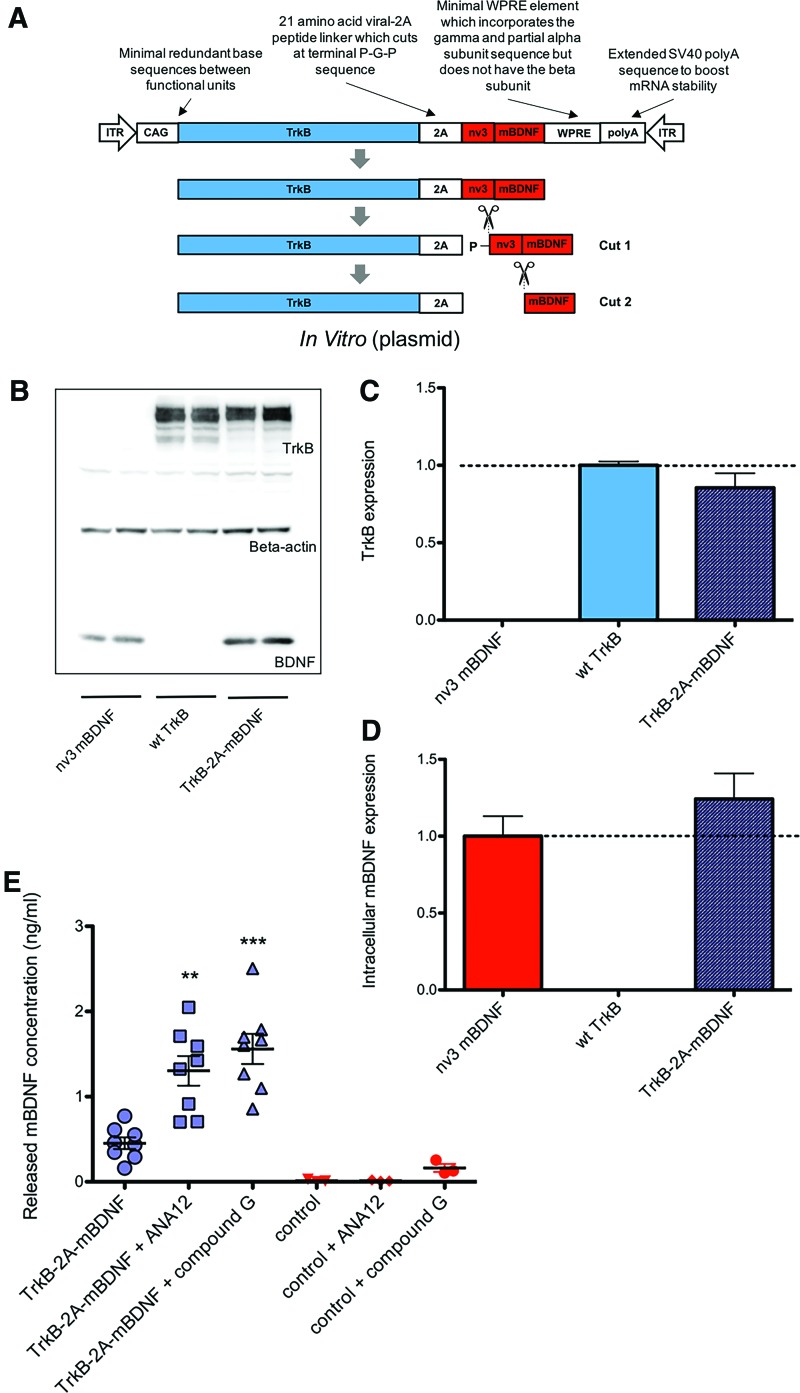
Processing of the plasmid TrkB-2A-mBDNF in HEK293 cells. **(A)** Schematic of the liberation of two mature proteins from a single transgene under the control of a single CAG promoter. **(B–D)** Correct cleavage and separation of proteins TrkB and BDNF compared to constructs expressing just BDNF (nv3 mBDNF) or TrkB (wild type TrkB) after 24 h (*n* = 4). **(E)** Release of BDNF could be detected from cells transduced with or without TrkB-2A-mBDNF after 24 h with the addition of the TrkB receptor antagonists ANA12 (10 μm) or compound G (10 μm) 1 h after transduction (*n* = 8); **p < 0.01 and ***p < 0.001 compared to TrkB-2A-mBDNF. Color images available online at www.liebertpub.com/hum

The next stage in the development of the gene therapy construct was to assemble the TrkB receptor with the optimal novel mBDNF construct, nv3 mBDNF. Plasmids containing wt TrkB were tested for the correct production, function, and translocation of the receptor (Supplementary Fig. S4) prior to incorporation into a dual construct, TrkB-2A-mBDNF (Fig. 3A).

Transduction of HEK293 cells with the plasmid TrkB-2A-mBDNF showed production of both TrkB and mBDNF at levels of protein equivalent to those generated by plasmids expressing the single transgenes (Fig. 3B–D). This highlighted that the large transgene was effectively cleaved intracellularly to generate two mature proteins. Examin-ation of the concentrations of secreted mBDNF following transduction with TrkB-2A-mBDNF was low (Fig. 3E), possibly indicating that most of the neurotrophin protein had been neutralized by binding to the TrkB receptors and subsequently internalized (which is necessary for long-term signal transduction). To confirm that mBDNF was initially released from transduced HEK293 cells, two potent TrkB receptor antagonists (ANA-12^40^ and compound G^41^) were added to the incubation medium shortly after transduction. As shown in Fig. 3E, there was a significant increase in the concentration of mBDNF measured in the culture medium compared to cells that did not contain each antagonist. This is consistent with the competitive blockade of the BDNF binding site at the TrkB receptor, thereby preventing internalization of the released mBDNF, and confirms that the neurotrophin is being fully separated from the TrkB receptor.

**Figure 4.**
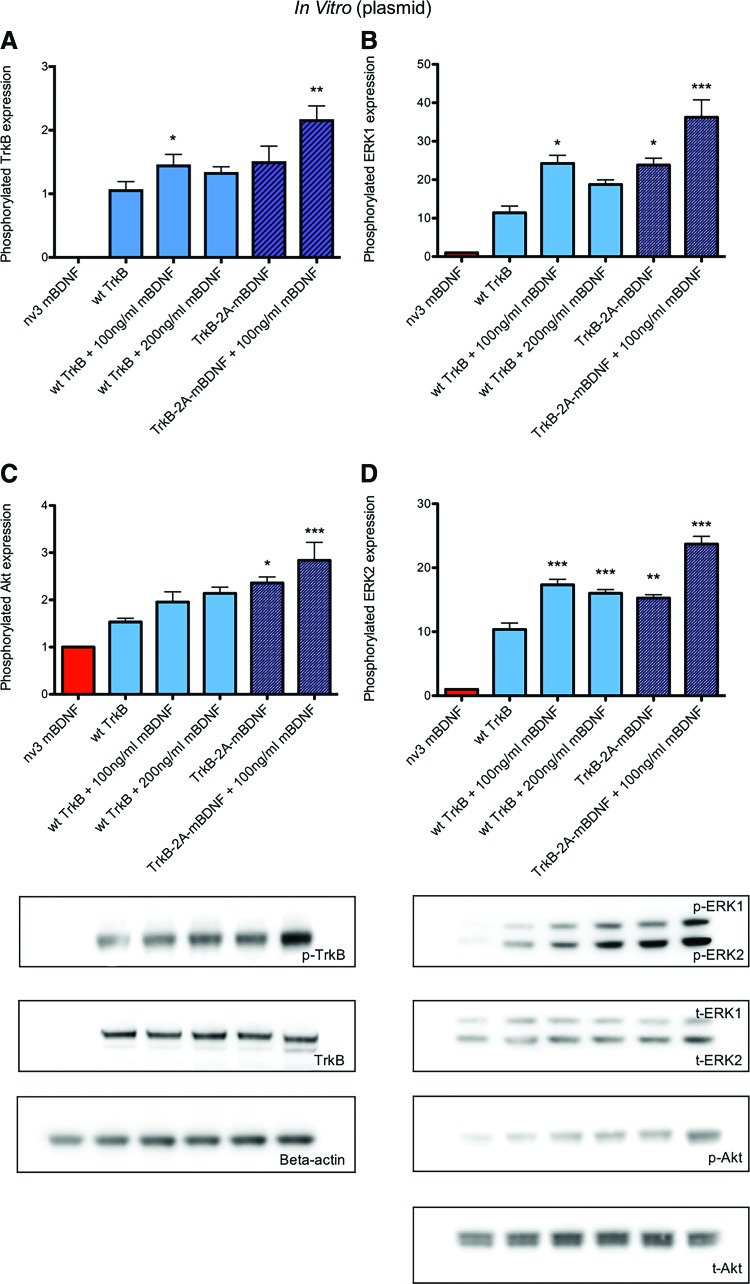
Downstream cell survival signaling pathways were upregulated in HEK293 cells 28 h after transduction with plasmid DNA + recombinant BDNF added for 4 h. Increased activation was also shown from HEK cells transduced with a construct expressing both the TrkB and BDNF ligand. **(A)** Phosphorylated (active) TrkB expression (*n* = 6) relative to total TrkB expression. **(B)** Phosphorylated (active) Akt expression (*n* = 6) relative to total Akt expression. **(C)** Phosphorylated (active) ERK1 expression total ERK1 expression (*n* = 6). **(D)** Phosphorylated (active) ERK2 expression relative to total ERK2 expression (*n* = 6); *p < 0.05, **p < 0.01, and ***p < 0.001 compared to nv3 mBDNF. Color images available online at www.liebertpub.com/hum

The effects of TrkB-2A-mBDNF transduction were then examined on two separate cell survival pathways downstream of TrkB phosphorylation. As shown in Fig. 4, there was a significant increase in the amount of phosphorylated (active) TrkB, Akt (p-Akt), and ERK-1/2 (p-ERK1 and p-ERK2) in the HEK293 cells lysates, indicating increased BDNF-TrkB receptor signaling. The density of p-Akt and p-ERK exceeded those achieved following transduction with plasmids producing only TrkB and were equivalent to the effects seen by adding 100–200 ng/mL recombinant BDNF to TrkB expressing HEK293 cells (Fig. 4B–D). Therefore, this study demonstrated that TrkB-2A-mBDNF could generate both TrkB receptors and mBDNF, which upon release was free to activate the receptors leading to increased intracellular cell survival signaling. Of interest was the fact that enhanced pathway activation could be detected after further application of recombinant BDNF (Fig. 4B–D), validating that the short 2A tag attached to the intracellular C terminus of the TrkB receptor was not interfering with kinase activity.

### Converting TrkB-2A-BDNF into an AAV2 vector

**Figure 5.**
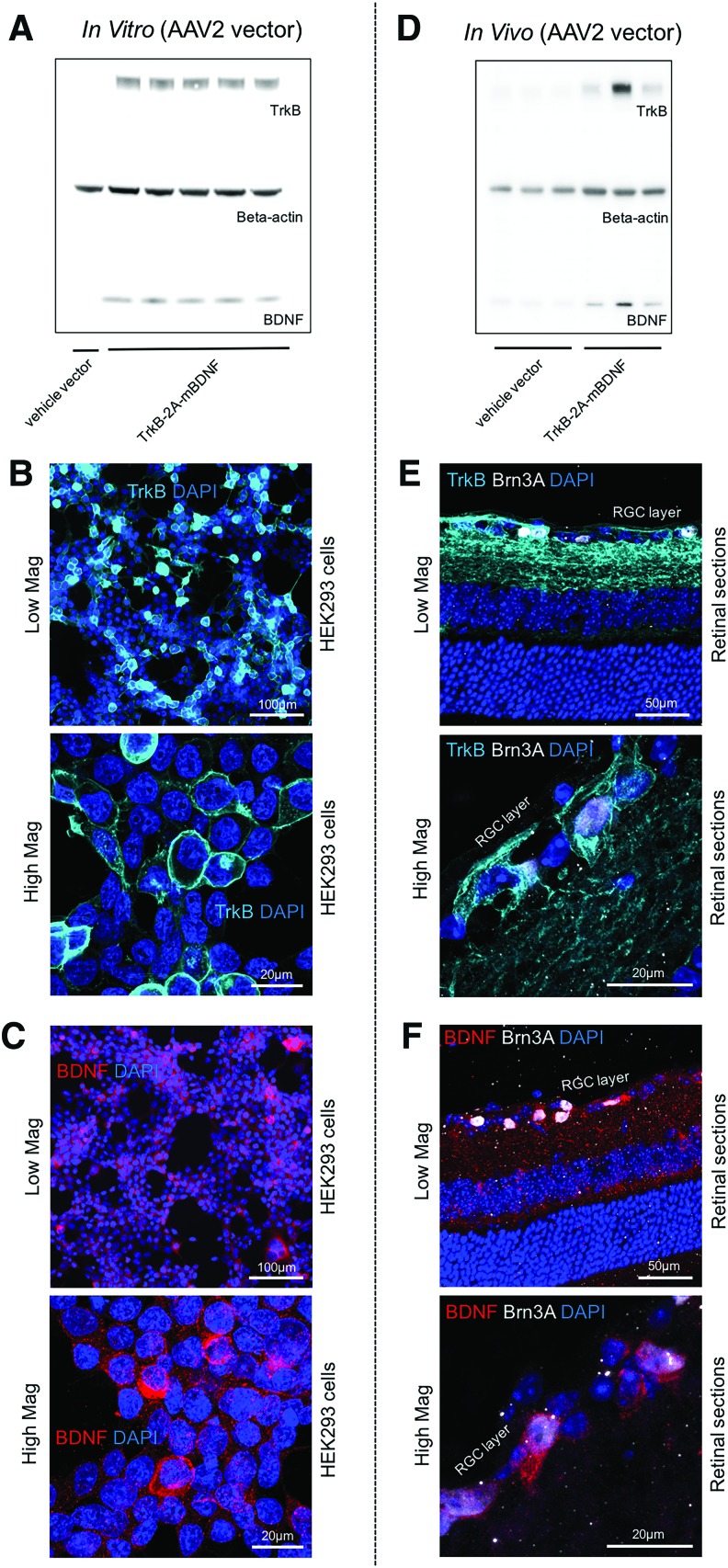
**(A)** HEK293 cells transfected with AAV2 vector TrkB-2A-mBDNF showed correct cleavage and separation of proteins TrkB and BDNF from a single transgene after 48 h (*n* = 5). **(B)** Immunocytochemistry revealed correct translocation to the membrane of TrkB, while **(C)** intracellular BDNF expression was observed in the cytosol of HEK293 cells. **(D)** *In vivo* transfection of mouse retina with AAV2 TrkB-2A-mBDNF increased TrkB and BDNF protein expression after 3 weeks (*n* = 4). **(E)** TrkB expression was detected on the cell surface of Brn3A+ RGCs and **(F)** BDNF within RGCs. AAV2 vectors were used at 1 × 10^10^ viral particles (vp)/mL *in vitro* and 1 × 10^10^ vp/eye *in vivo*. Color images available online at www.liebertpub.com/hum

The TrkB-2A-mBDNF transgene was then incorporated into an AAV2 vector (Supplementary Fig. S1) and tested in HEK293 cells to confirm transfection and construct processing. TrkB receptors and BDNF were correctly translated and cleaved at the expected molecular weights (Fig. 5A). Immunocytochemical analysis showed a large proportion of HEK293 cells were transfected with the AAV2 construct and that BDNF was detected intracellularly while TrkB receptors translocated to the cell membrane (Fig. 5B and C). Testing the AAV2 TrkB-2A-mBDNF vector within the rodent eye also confirmed correct separation of the ligand and receptor and translocation to the correct compartments of RGCs *in vivo* (Fig. 5D–F).

### Neuroprotective effect of AAV2 TrkB-2A-mBDNF

**Figure 6.**
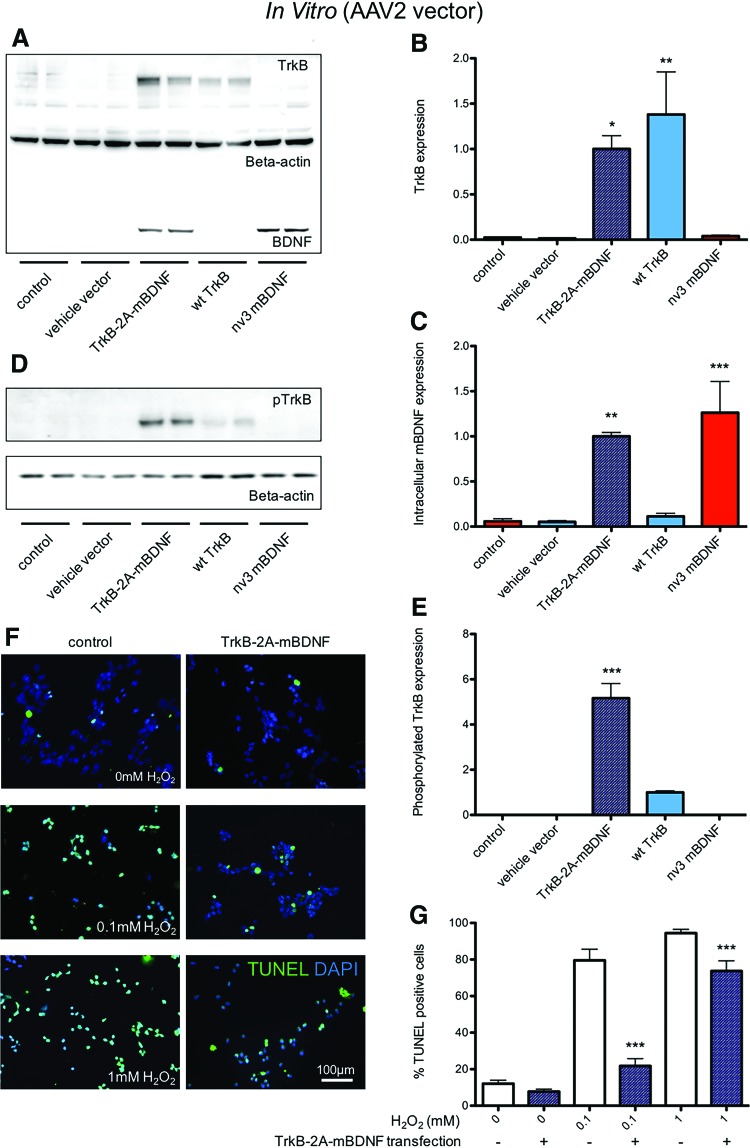
SH-SY5Y cells were transfected with AAV2 vectors to increase the expression of BDNF, TrkB, or both proteins. **(A)** Correct cleavage and separation of proteins TrkB and BDNF was detected 48 h after transfection (*n* = 4). **(B** and **C)** Comparable protein expression from the dual construct AAV2 TrkB-2A-mBDNF was detected compared to individual, single AAV2 constructs (*n* = 4); *p < 0.05, **p < 0.01, and ***p < 0.001 compared to controls. **(D** and **E)** Increased phosphorylated (active) TrkB relative to total TrkB was measured in cell lysates of AAV2 TrkB-2A-mBDNF transfected cells compared to AAV2 wt TrkB (*n* = 4); ***p < 0.001. **(F** and **G)** Transfection with AAV2 TrkB-2A-mBDNF could reduce the number of apoptotic (*green*) cells following treatment with H_2_O_2_ for an additional 24 h after transfection (*n* = 6); ***p < 0.001 compared to non-transfected cells at the same concentration H_2_O_2_. All AAV2 vectors were used at 1 × 10^10^ viral particles (vp)/mL. Color images available online at www.liebertpub.com/hum

To test the neuroprotective ability of BDNF-TrkB receptor signaling in a neuronal cell line, immature SH-SY5Y human neuroblastoma cells were transfected with AAV2 vectors expressing nv3 mBDNF, wt TrkB, or the dual construct TrkB-2A-mBDNF. Transfection resulted in high levels of TrkB and mBDNF expression after 48 h (Fig. 6A). Like plasmid and AAV2 vector transfection in HEK293 cells, effective 2A cleavage occurred in SH-SY5Y cells transfected with AAV2 TrkB-2A-mBDNF (Fig. 6B and C). Phosphorylated (active) TrkB receptors were observed in SH-SY5Y cells transfected with the Trk3B-only AAV2 vector (due to auto-phosphorylation), and further, enhanced activation was observed with AAV2 TrkB-2A-mBDNF, indicating a beneficial effect to producing BDNF in combination with its receptor (Fig. 5A and D). Exposing SH-SY5Y cells to H_2_O_2_ (at either 0.1 or 1.0 mM) produced oxidative damage leading cell apoptosis, measured by TUNEL staining (Fig. 5F and G). Transfection of the SH-SY5Y cells with AAV2 TrkB-2A-mBDNF prior to H_2_O_2_ exposure could significantly attenuate cellular loss by apoptosis. These data indicated that the expression of both TrkB and mBDNF can impart significant neuroprotective capability to SH-SY5Y cells, thereby preventing cell death when exposed to physiological stress.

## Conclusions

Maintained activity of the mBDNF/TrkB neurotrophin signaling pathway has been proposed as a powerful therapeutic solution to prevent neuronal cell loss in several neurological conditions with diverse pathologies.^9,23,47,48^ However, simple administration of recombinant mBDNF, or increased BDNF production through gene therapy, are both limited by the downregulation and loss of the TrkB receptor at the target cells, thereby significantly attenuating any therapeutic benefits.^30^ Moreover, in many neurological conditions, there are reports of significant diminution in TrkB density.^21,25,48^

Previous research has shown that overexpression of TrkB receptors provides significant neuroprotection to RGCs in animal models of optic nerve injury and that further efficacy could be achieved when coupled with ligand administered as recombinant protein.^47^ It was therefore deduced that a gene therapy capable of generating both the TrkB receptor and its ligand might overcome the tachyphylaxis and provide autocrine signaling, as can be found in the hippocampus,^49^ and paracrine signaling on adjacent cells.

However, the coding sequences for TrkB and BDNF would be too large to accommodate within an AAV vector as a conventional bicistronic construct (Supplementary Fig. S1). Therefore, the decision was made to alter the BDNF sequence and utilize a viral-2A peptide sequence for separation of the two transgenes. Omitting the coding sequence for proBDNF both reduced the size of the construct and removed the potentially pro-apoptotic effects of proBDNF production, which leads to exacerbation of β-amyloid neurotoxicity^20^ and activation of p75^NTR^ receptors.^17^ Additionally, in neuronal cells, mBDNF has been proposed to be the predominant form of BDNF^13,14^ and therefore would remain the principal form in this system.

It should be noted that removal of the pro-domain has the potential to interfere with the intracellular binding to sortilin, which facilitates its transportation from the Golgi complex to dense core secretory vesicles^15^ in addition to having the potential to reduce protein folding.^10,11^ However, substitution with a novel signal peptide sequence appeared to circumvent the sortilin-directed transport pathway, directing the protein into the constitutive secretory pathway.^15^ Lou *et al.*^50^ previously demonstrated that it was possible to direct BDNF containing the pro-domain from the sortilin-regulated secretory pathway to the constitutive secretory pathway by mutating the acidic residues of mBDNF (E146 and D234) to alanines. It is therefore possible that in the absence of binding to sortilin within the Golgi complex, the mBDNF sorting by carboxypeptidase E facilitates the protein to be transported via the alternative constitutive secretory pathway.

The introduction of the viral-2A peptide sequence ensured correct processing and translocation of the TrkB receptor and mBDNF coding element *in vitro* in human HEK293 and SH-SY5Y cells and *in vivo* within RGCs of the mouse eye. The dual construct also demonstrated an added benefit to expressing both ligand and receptor compared to individual constructs with substantially upregulated cell survival pathways and the ability to provide neuroprotection against hydrogen peroxide oxidative damage.

In conclusion, a gene therapy construct has been designed that aims to provide long-term signaling via the BDNF-TrkB pathway and neuroprotection against a variety of pathophysiological insults that are encountered in neurological diseases ranging from glaucoma to Alzheimer's disease. This construct is now being tested in animal models of neurological disease to examine for beneficial effects on neuronal survival and function and assess the potential for further development toward human clinical trials.

## Supplementary Material

Supplemental data

Supplemental data

Supplemental data

Supplemental data

## Notes

^*^ www.idtdna.com/CodonOpt
