# Supplementary material for: Design of a Novel Gene Therapy Construct to Achieve Sustained Brain-Derived Neurotrophic Factor Signaling in Neurons
Source: Hum Gene Ther. 2018 Jul 1;29(7):828–41. doi: 10.1089/hum.2017.069 (PMC6066195; doi:10.1089/hum.2017.069)
Supplement: Supplemental data [file Supp_Fig1.pdf]

Supplementary Data

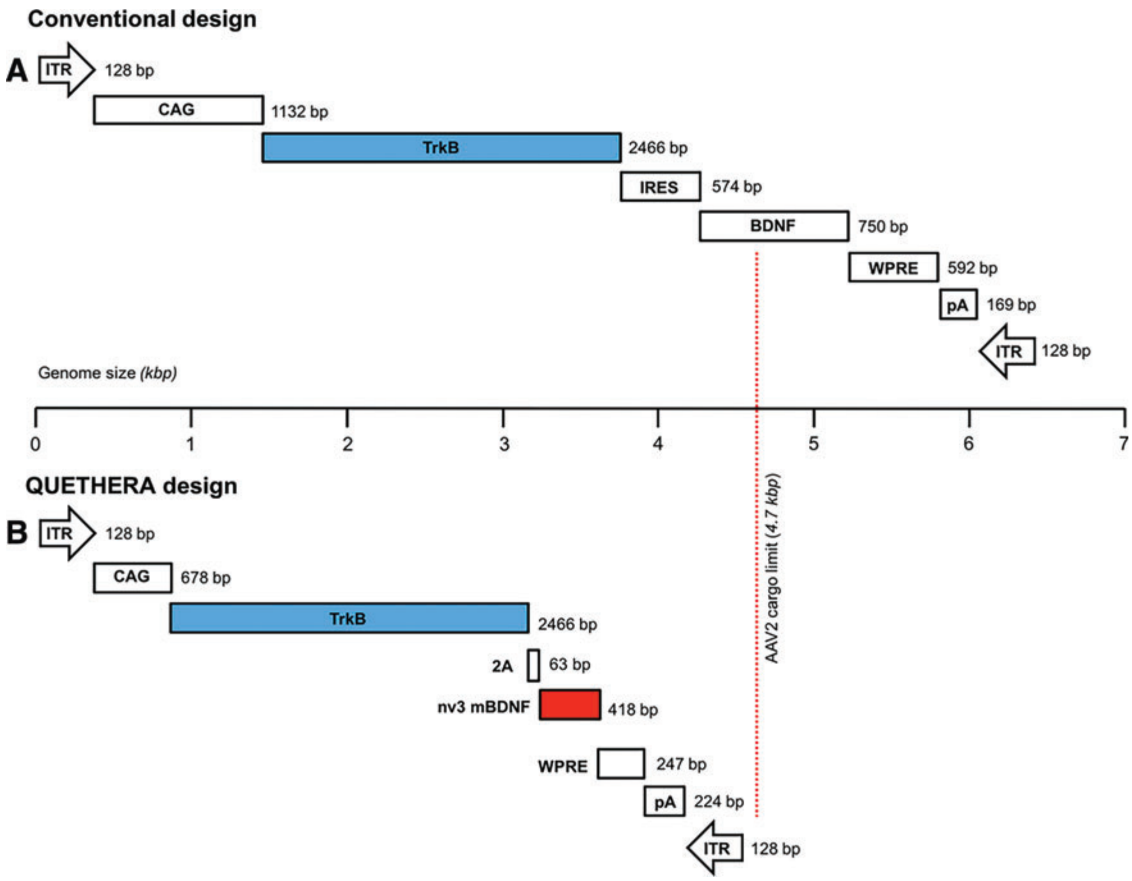

Supplementary Figure S1. (A) Schematic representation of the conventional elements necessary for production of TrkB and BDNF in an AAV2 vector system. **(B)** Schematic representation of elements used in the novel AAV2 TrkB-2A-mBDNF construct tested in this study. BDNF, brain-derived neurotrophic factor; ITR, inverted terminal repeat; IRES, Internal Ribosome Entry Site; mBDNF, mature BDNF; WPRE, woodchuck hepatitis virus posttranscriptional regulatory element; pA, polyadenylation sequence.
