# Supplementary material for: Design of a Novel Gene Therapy Construct to Achieve Sustained Brain-Derived Neurotrophic Factor Signaling in Neurons
Source: Hum Gene Ther. 2018 Jul 1;29(7):828–41. doi: 10.1089/hum.2017.069 (PMC6066195; doi:10.1089/hum.2017.069)
Supplement: Supplemental data [file Supp_Fig2.pdf]

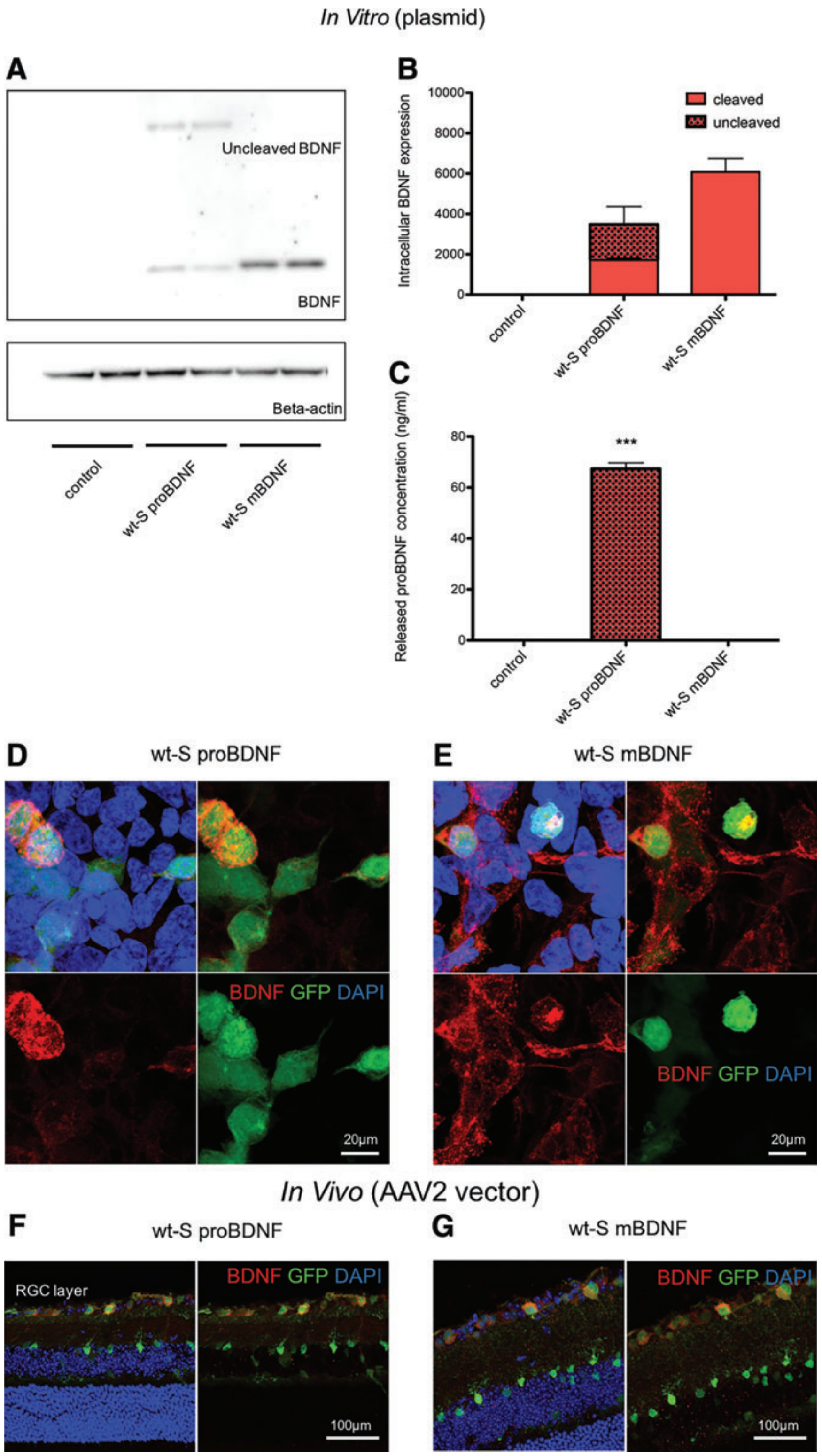


**Supplementary Figure S2.** Comparison of HEK293 cells transduced or mouse retina transfected with constructs expressing wild-type BDNF (wt-S proBDNF) or a construct absent of BDNF's pro-domain (wt-S mBDNF). **(A** and **B)** Protein expression for both cleaved (mature) and uncleaved (pro) BDNF could be detected in wt-S proBDNF, while uncleaved BDNF was absent in wt-S mBDNF 24 h after transduction in HEK293 cells ($n = 4$). **(C)** Secreted, uncleaved BDNF was only observed in wt-S proBDNF culture medium 24 h after transduction ($n = 4$); ***$p < 0.001$ compared to controls. **(D** and **E)** Only transduced, GFP-positive, HEK293 cells produced BDNF with similar intracellular localization between constructs. **(F** and **G)** AAV2 vector transfected, GFP-positive, retinal cells expressed BDNF with greatest BDNF production in transfected retinal ganglion cell (RGC) layer neurons. AAV2 vectors were used at $5.8 \times 10^9$ viral particles (vp)/eye. AAV, adeno-associated virus; GFP, green fluorescent protein.
