# Supplementary material for: Design of a Novel Gene Therapy Construct to Achieve Sustained Brain-Derived Neurotrophic Factor Signaling in Neurons
Source: Hum Gene Ther. 2018 Jul 1;29(7):828–41. doi: 10.1089/hum.2017.069 (PMC6066195; doi:10.1089/hum.2017.069)
Supplement: Supplemental data [file Supp_Fig3.pdf]

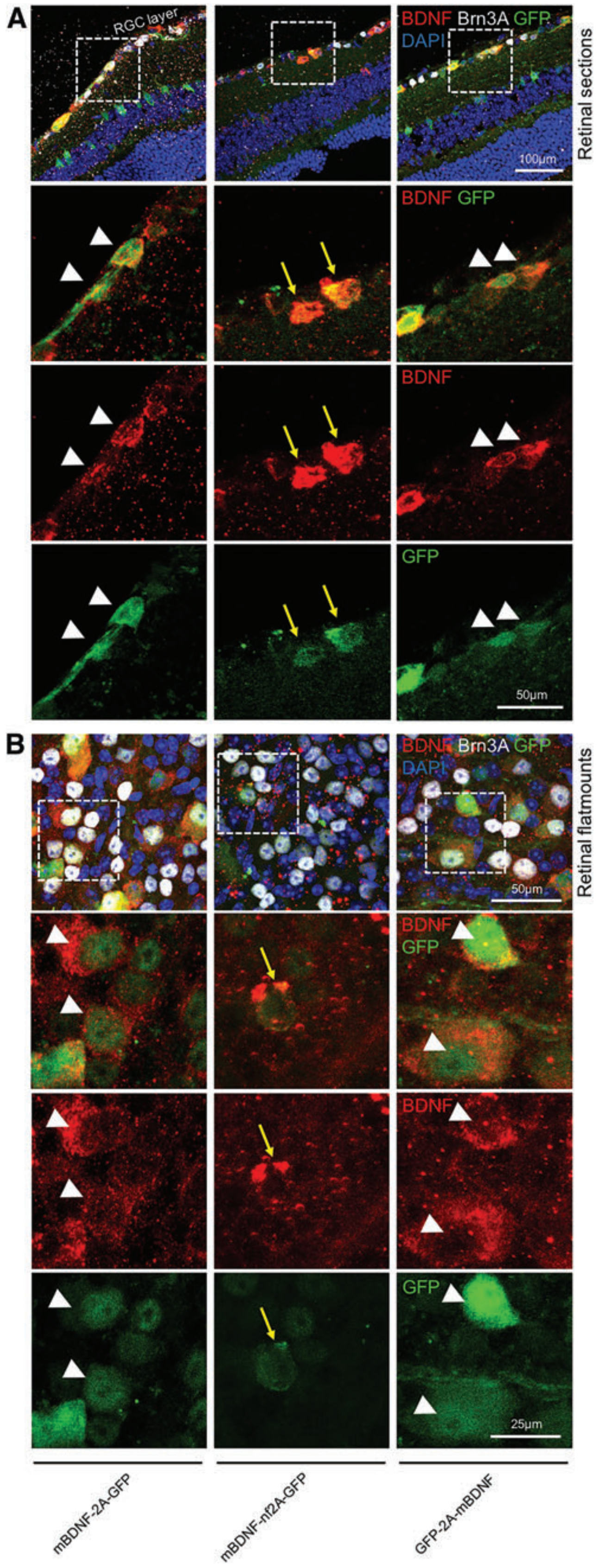

Supplementary Figure S3. Cleavage and separation of GFP and BDNF *in vivo* in the mouse retina 3 weeks after AAV2 vector transfection. **(A** and **B)** GFP and BDNF could be detected in separate locations (*white arrows*) when the 2A viral linker was functional, while use of a non-functional cleavage site did not separate proteins (*yellow arrows*). All AAV2 vectors used at 1×10^{10} viral particles (vp)/eye.
