# Supplementary material for: Design of a Novel Gene Therapy Construct to Achieve Sustained Brain-Derived Neurotrophic Factor Signaling in Neurons
Source: Hum Gene Ther. 2018 Jul 1;29(7):828–41. doi: 10.1089/hum.2017.069 (PMC6066195; doi:10.1089/hum.2017.069)
Supplement: Supplemental data [file Supp_Fig4.pdf]

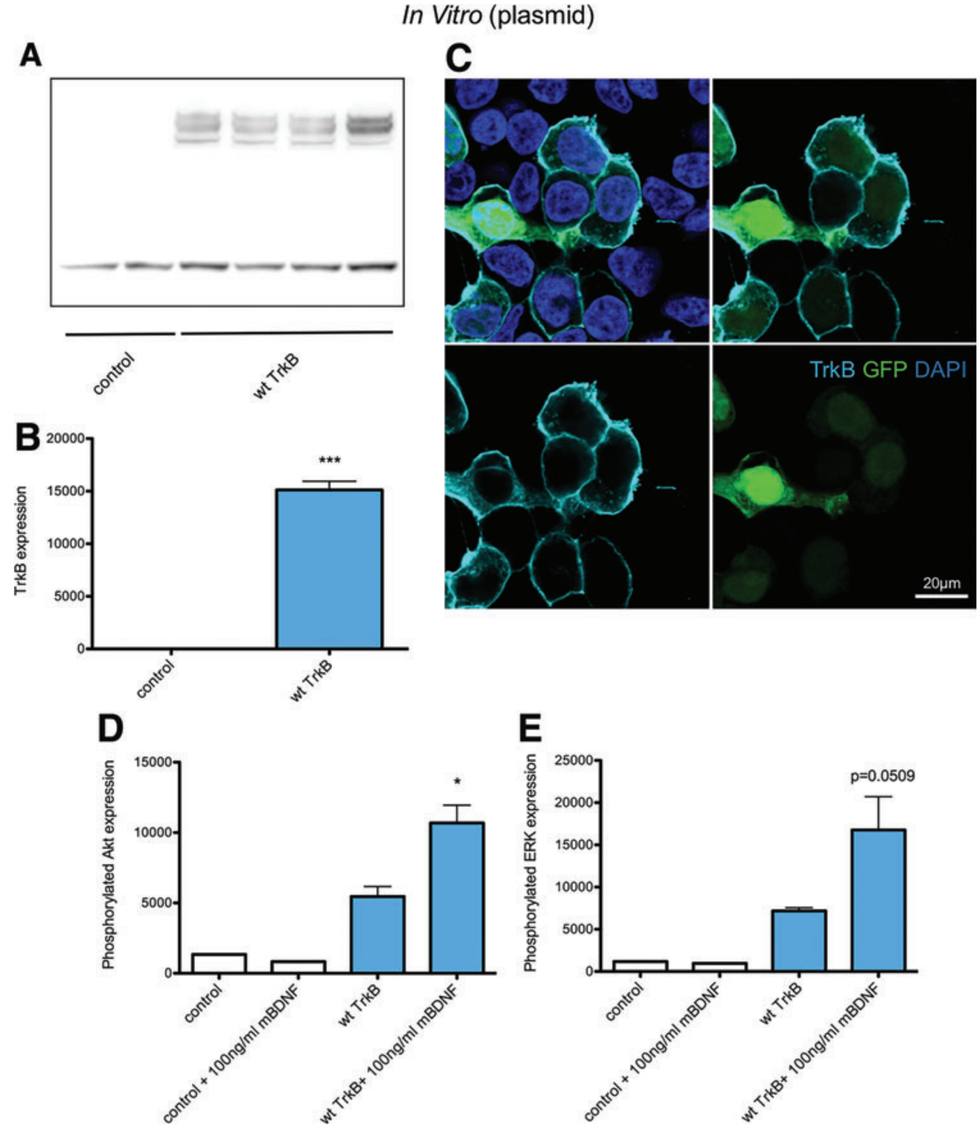

Supplementary Figure S4. Independent testing of HEK293 cells transduced with wt TrkB ensured the transgene for TrkB was correct and functional prior to incorporation into construct TrkB-2A-mBDNF. **(A** and **B)** HEK293 cells expressed TrkB protein 24 h after transduction with wt TrkB ($n=4$); $p<0.001$ compared to control. **(C)** Expression of the TrkB receptor was observed around the cell membrane of transduced, GFP-positive cells. **(D** and **E)** Downstream activation could be detected after administering recombinant mBDNF for 4 h to TrkB expressing HEK293 cells ($n=4$); $*p<0.05$ compared to wt TrkB. TrkB, tropomyosin-related receptor-B.
